# The ecological footprint of medical AI

**DOI:** 10.1007/s00330-023-10123-2

**Published:** 2023-08-15

**Authors:** Daniel Truhn, Gustav Müller-Franzes, Jakob Nikolas Kather

**Affiliations:** 1 Department of Diagnostic and Interventional Radiology, University Hospital RWTH Aachen, Aachen, Germany; 2 Else Kroener Fresenius Center for Digital Health, Technical University Dresden, Dresden, Germany; 3 Department of Medicine I, University Hospital Dresden, Dresden, Germany; 4 Medical Oncology, National Center for Tumor Diseases (NCT), University Hospital Heidelberg, Heidelberg, Germany

Artificial intelligence (AI) is progressively being woven into the fabric of clinical medicine. Most notably, radiology, as an image-based medical specialty, offers numerous applications of AI, including image reconstruction, image analysis, and clinical decision-making. A rich body of research shows the potential of AI in radiology workflows, and indeed, many AI-based medical devices are already available and have received regulatory approval in major markets for clinical routine use. Existing AI models can support radiologists in a wide range of tasks, reaching from image acquisition [1] to diagnosis [2] and outcome prediction [3]. In the future, the development of foundation models, i.e., large multimodal transformer neural networks that can manipulate text, images, and other data types, will conceivably increase the use of AI in radiology and medicine even more [4]. The advancements in large language models (LLMs), e.g., ChatGPT, are a testament to these technical developments, as these LLMs already have the capability to reason about clinically relevant topics across a wide range of domains.

Behind this effectiveness of contemporaneous AI models are two primary resources: access to large high-quality datasets and the ability to train huge neural networks with up to hundreds of billions of parameters. As these neural networks are trained and are being deployed at scale, a high computational demand is exerted, which consumes electricity and hence leaves a carbon footprint.

With the alarming verdict from the Intergovernmental Panel on Climate Change (IPCC-2021) suggesting that global warming of 1.5 °C and 2 °C will be surpassed this century unless drastic CO_2_ and other greenhouse gas emissions reductions are undertaken, we cannot overlook the energy and carbon costs involved in developing and using AI models in medicine. According to the United Nations Sustainable Development Goals Report 2022, taking urgent action to combat climate change is a top priority for our societies and the key instrument is to reduce and in the near future completely remove any carbon emissions.

How big a problem are medical AI models in this regard? To help comprehend the scale of this ecological impact, let us examine some relatable comparisons. On an annual basis, an average inhabitant of EU-27 contributes about 6.8 tons of CO_2_ emissions, as per greenhouse gas emission statistics [5]. A round-trip flight between Munich and New York in economy class emits around 2.1 tons of CO_2_ per person, according to Stiftung Myclimate [6]. And if we look at medical equipment, an MRI scanner running for 1 year results in around 58.3 tons of CO_2_ emissions which amounts to 14.6 kg CO_2_ per scan for a utilization of 4000 scans per year [7].

Consider, now, the carbon footprint of training a large language model like GPT-3, the predecessor of the first ChatGPT model. Training such an LLM leaves the same carbon footprint of roughly 262 persons taking a round trip flight from Munich to New York or running one MRI machine in a typical hospital environment for 9.5 years [8]. Comparatively, the carbon emission of conversing with a mid-sized language model in 20 interactions (i.e., giving 20 prompts) typically is equivalent to about a millionth of a one-person round trip transatlantic flight [8]. Of note, this number depends on multiple factors, such as the size of the language model, or the infrastructure used for deployment, but we estimate, that this number is correct within few orders of magnitude even for larger contemporary language models. Let us examine a more specific example from medical research, where smaller AI models are frequently used. Consider a state-of-the-art network undergoing training on a large database comprising hundreds of thousands of images for 100 hours. This equates roughly to the typical daily carbon footprint of a European citizen [9]. This is modest. In the future, when AI models will increasingly be applied the carbon footprint of deploying the model will be more relevant. Let us assume that an AI model is run on the images of a patient that has just undergone an MR examination. In our experience, applying a model on a new examination takes a fraction of a second, but to account for bigger models in the future, we estimate that the model will occupy a state-of-the-art GPU in the cloud for at most 10 s. This amounts to a carbon footprint of 0.5 g [9]. Compare this to the carbon emission for the MRI scan itself which amounts to 14,600 g [7].

The magnitude of this carbon footprint seems modest, although, with AI models poised to play a central role in tomorrow’s healthcare at a massive scale, these small environmental impacts can add up to relevant amounts and thus represent an opportunity for improvement.

We, as medical professionals, including radiologists, can have an influence by our purchase decisions and by proxy on the model providers. This can push the industry towards reducing the computational load for AI models. Models can be condensed to improve efficiency. The utilization of large, centralized data centers can offer up to twice the efficiency due to factors like optimized computational time usage and efficient cooling system [10]. Moreover, specialized hardware optimized for neural network inference could further reduce energy consumption. A transition towards clean energy sources for data centers is inevitable to remove carbon emissions. This is also economically reasonable, as wind and solar power are much cheaper than fossil fuel-based electricity.

Still, these actions represent changes at a small level, and often at an individual level. The magnitude of greenhouse gas emissions, the urgency of mitigating climate change, and the existential threat to our planet demand collective action. The healthcare sector, where we as medical doctors exert a considerable influence, is a relevant part of our society that could help to shift society on a sustainable path. An important area where such influence can be employed is investment decisions—be it on a personal level, or through indirect control and lobbying on a collective level. Large sums of money of medical professionals are for example tied up in pension funds. If we consciously decide to steer these investments away from fossil fuels and towards sustainable industries the positive impact on our planet could be substantial. This action, known as divestment, could result in reduced carbon emissions. In fact, divestment symbolizes the type of collective actions that can bring about tangible change, but there are others such as using the considerable trust society places in healthcare professionals and engaging collectively in initiatives such as the Physicians for Social Responsibility (http://www.psr.org) to spread awareness of the problem and potential solutions.

In conclusion, while AI’s role in radiology and its associated carbon footprint are indeed relevant topics, the potential for more substantial impact goes beyond individual actions. AI in healthcare is poised to grow, but alongside its development, we must take conscious, collective steps towards sustainability at a larger scale. By incorporating these considerations into our professional and financial decisions, we can contribute to a more sustainable healthcare system and a liveable planet for future generations.

## Declarations

### Guarantor

The scientific guarantor of this publication is Daniel Truhn.

### Statistics and biometry

No complex statistical methods were necessary for this paper.

### Methodology

• Commentary
